# A Rare Case of Paraganglioma Syndrome Associated With Polycythemia and Blindness

**DOI:** 10.7759/cureus.63047

**Published:** 2024-06-24

**Authors:** Yumiko Esaki, Xiaoyan Liao, Inga Harbuz-Miller

**Affiliations:** 1 Department of Medicine, Division of Endocrinology, Diabetes and Metabolism, University of Rochester Medical Center, Rochester, USA; 2 Department of Pathology and Laboratory Medicine, University of Rochester Medical Center, Rochester, USA

**Keywords:** pacak-zhuang syndrome, hif-2α, epas-1, paraganglioma, pheochromocytoma

## Abstract

Pheochromocytomas and paragangliomas (PPGL) are rare neuroendocrine tumors. They can be diagnosed independently or as part of a syndrome, especially with germline mutations. Rarely, a somatic mutation can present as part of a syndrome associated with recurrent PPGL, congenital polycythemia, and vascular malformation. We report a case of a 44-year-old man with a history of congenital blindness, stroke in utero, cerebral ataxia, and polycythemia since age 12, treated with phlebotomies who presented with back pain and hypertension. Abdominal computer tomography with IV contrast showed a right adrenal enhancing lesion measuring 1.4 x 1.2 cm and a conglomerate of heterogeneously enhancing periaortic lesions measuring up to 5 cm in the mid-abdomen. Biochemical workup revealed plasma free normetanephrine 27.5 nmol/L (0.00-0.89) and plasma free metanephrine 0.49 nmol/L (0.00-0.49). Histopathology confirmed synchronous pheochromocytoma and paraganglioma. This case illustrates the importance of taking a detailed past medical history and the relevance of polycythemia in the paraganglioma workup.

## Introduction

Pheochromocytomas and paragangliomas (PPGL) are rare neuroendocrine tumors originating from neural crest cells in the adrenal medulla and in extra-adrenal sympathetic and parasympathetic paraganglia, respectively, with an annual incidence of 500-1600 cases per year in the United States [1]. Approximately 30-35% of PPGLs harbor germline mutations while 35-40% are due to somatic mutations [2]. These mutations are subdivided into three main clusters: cluster 1, or pseudohypoxia cluster, cluster 2, or kinase-signaling cluster, and cluster 3, or Wnt signaling cluster. Cluster 1 is characterized by activation of pathways that mimic hypoxia signaling. This cluster is further subdivided into clusters 1A and 1B. Cluster 1A includes Krebs cycle-related genes (e.g., succinate dehydrogenase subunit *(SDH)* genes such as *SDHA*, *SDHB*, *SDHC*, and *SDHD*) and cluster 1B includes Von-Hippel Lindau *(VHL)* and endothelial PAS domain protein 1 *(EPAS-1)* genes. Recent studies have identified the association between somatic mutations of the *EPAS-1* gene encoding hypoxia-inducible factor 2 alpha (HIF-2α) and a new syndrome of PPGL associated with congenital polycythemia, called Pacak-Zhuang syndrome [3-5]. Patients with this syndrome can present with recurrent PPGL, somatostatinoma [4,6], congenital malformation of intracranial veins [7], and malformation of the macula and retina [8]. Herein, we present a case with blindness, polycythemia, and multifocal PPGL with suspected Pacak-Zhuang syndrome. This case illustrates the challenges of diagnosis in this syndrome and the limitation of identifying the somatic mutations.

This case was previously presented as a poster abstract at the 2022 ENDO Meeting on June 11, 2022.

## Case presentation

A 44-year-old man with a complex past medical history, including in utero stroke, congenital blindness, and polycythemia treated with phlebotomies since age 12 was found to have an incidental adrenal mass and a conglomerate of periaortic lesions during the imaging evaluation for chronic back and abdominal pain. On a detailed review of systems, he endorsed symptoms of episodic palpitations associated with diaphoresis, chronic anxiety, headaches, and hypertension for over eight years. He noticed unintentional weight loss of about 16% of his body weight over the six months before presentation. No known family history of malignancy or hereditary syndromes was reported.

Diagnostic assessment

**Figure 1:**
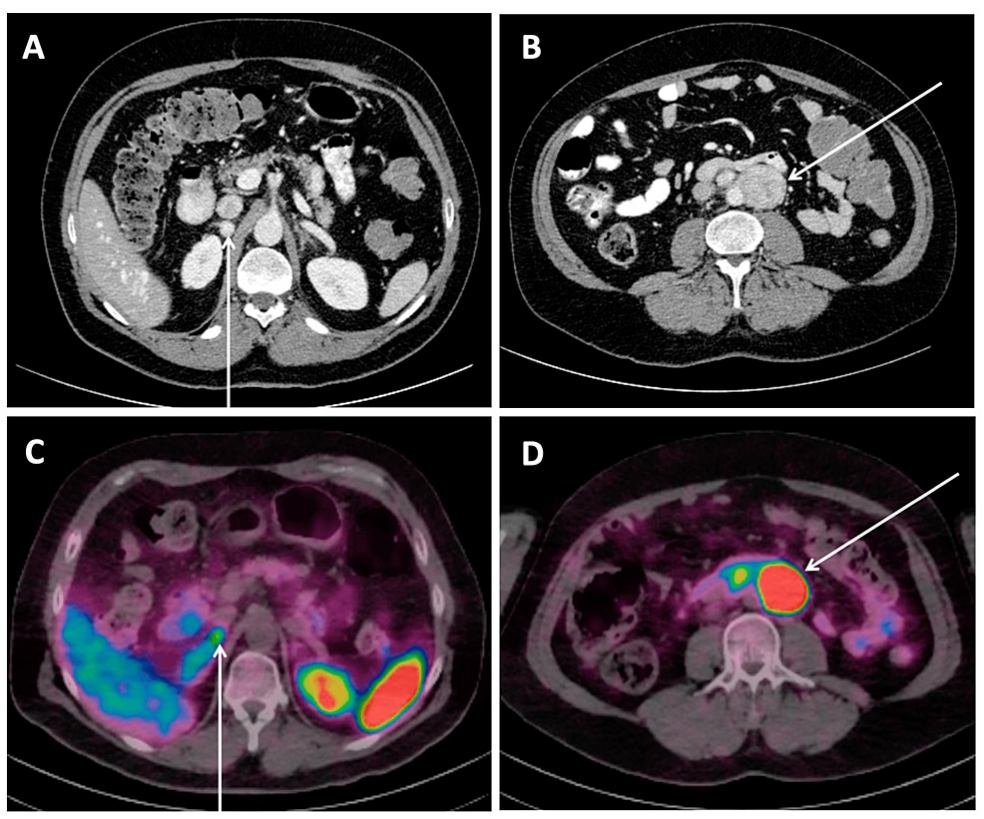
Computed tomography of the abdomen with IV contrast (A, B) and Gallium 68 DOTATATE positron emission tomography CT (C, D) revealing a right adrenal lesion(A, C) and a conglomerate of periaortic lesions(B, D) CT abdomen with contrast showing a right adrenal enhancing lesion measuring 1.4 x 1.2 cm, 190 Hounsfield units post-contrast (A, arrow), and a conglomerate of heterogeneously enhancing periaortic lesions measuring up to 5 cm in the mid-abdomen (B, arrow). In the Gallium 68 DOTATATE PET-CT, a corresponding avid right adrenal nodule (C, arrow) and a retroperitoneal lesion (D, arrow) were demonstrated.

Following the current guidelines for the diagnostic assessment of an adrenal incidentaloma and pheochromocytoma [9,10], biochemical testing ruled out hypercortisolemia and primary hyperaldosteronism. Plasma free metanephrines by liquid chromatography-mass spectrometry (LCMS) revealed the normetanephrine to be elevated 30 times above the normal range at 27.5 nmol/L (0.00-0.89) and plasma free metanephrines of 0.49 nmol/L (0.00-0.49). A contrast-enhanced abdominal computer tomography (CT) obtained for abdominal pain revealed a right adrenal enhancing lesion measuring 1.4 x 1.2 cm, 190 Hounsfield units post-contrast, and a conglomerate of heterogeneously enhancing periaortic lesions measuring up to 5 cm in the mid-abdomen (Figures 1A, 1B). The washout and pre-contrast radiodensity were not reported, given that this study was not a dedicated adrenal protocol scan. A Gallium 68 DOTATATE scan showed multiple avid retroperitoneal lesions measuring up to 4.4 cm and an avid right adrenal nodule corresponding to the lesions found on the CT scan (Figures 1C, 1D).

Treatment

In preparation for surgical resection, an alpha blockade with doxazosin was initiated and titrated up to 8 mg daily. Due to reflex tachycardia and persistent hypertension, diltiazem was added for optimal blood pressure and heart rate control. The patient underwent a right adrenalectomy and periaortic mass resection. Postoperatively, his symptoms, including episodic diaphoresis associated with headaches, tachycardia, palpitation, and anxiety, resolved. He was discharged home off all antihypertensives.

Outcome and follow-up

**Figure 2:**
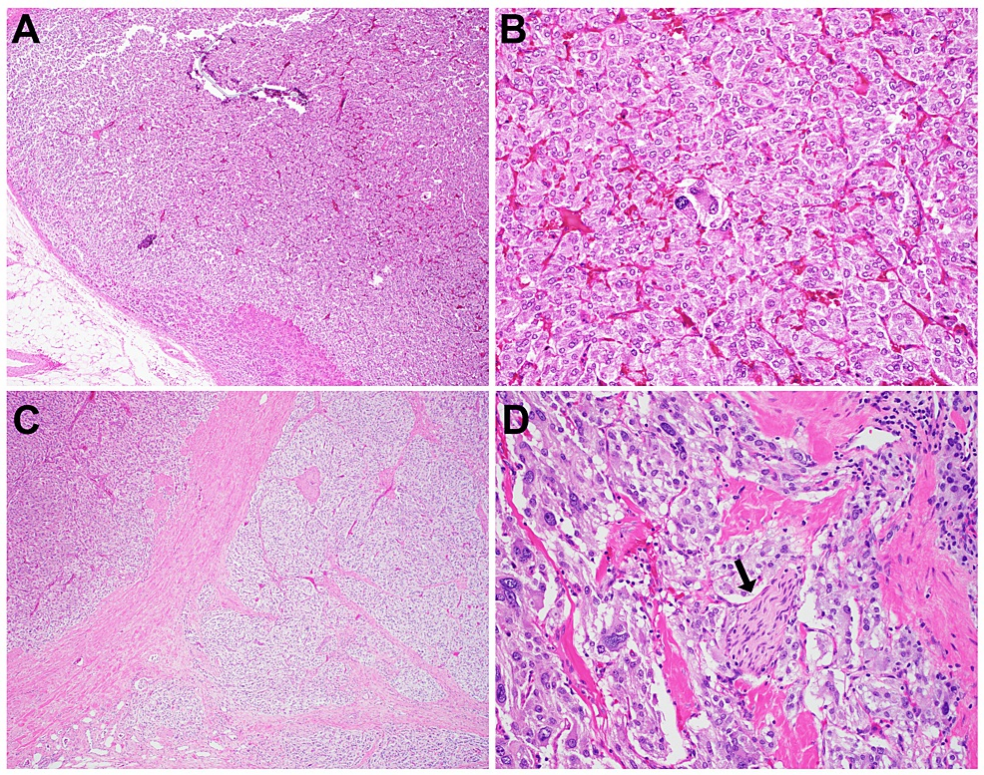
Histology of the pheochromocytoma (A-B) and abdominal retroperitoneal paraganglioma (C-D). (A) In the adrenal gland, the pheochromocytoma is confined with a rim of normal cortical tissue (lower left, A). The tumor shows focal cytologic atypia (B). The retroperitoneal abdominal paraganglioma demonstrates infiltrative growth (C) and increased cytologic atypia (D). Perineural invasion (D, arrow) and lymphovascular invasion with one lymph node metastasis are also noted (not shown). Magnifications: A and C: 40x; B and D: 200x

The histopathologic examination of the surgical specimen confirmed synchronous pheochromocytoma and paraganglioma (Figures 2A-2D). Grossly, the pheochromocytoma measured 1.5 cm and was well-circumscribed with a Ki-67 labeling index < 1%, without lymphovascular invasion or tumor necrosis. There was a capsular and periadrenal fat invasion. The paraganglioma appeared multinodular and measured 9.3 cm, with a Ki-67 labeling index < 1% and clear margins. Lymphovascular invasion was noted. One out of nine lymph nodes revealed positive metastatic disease.

Germline mutation testing was negative for suspected polycythemia/PPGL syndrome and other hereditary PPGL using the Invitae 14 gene panel, which included *MAX*, *NF1*, *RET*, *SDHA*, *SDHAF2*, *SDHB*, *SDHC*, *SDHD*, *TMEM127*, *VHL*, *EGLN1*, *FH*, *KIF1B*, and *MEN1*. Additionally, the germline *EPAS-1* single gene analysis was negative. Genetic testing of the tumor tissue was obtained via the Tempus xT 648 gene panel, a next-generation sequencing (NGS)-based cancer genome profiling test. It interrogates 648 cancer-related genes in tumor tissues. The assay can detect clinically relevant alterations as well as potential germline findings. The test was negative except for two variants of unknown significance (*SLIT2*, *MYCN*). Unfortunately, the Tempus panel does not include the *EPAS-1* gene.

## Discussion

This case illustrates a rare syndrome of PPGL, polycythemia, and congenital blindness, likely due to a somatic mutation of *EAPS-1*. Activating mutations of the *EPAS-1* gene lead to the stabilization of the HIF-2α protein, which functions as a transcription factor upon dimerization with the HIF-2β protein [3]. This results in the transcription of hypoxia-inducible genes, leading to angiogenesis, polycythemia, and tumorigenesis. Most patients present with early childhood polycythemia, PPGL in later years, with or without associated vascular formation of the eyes and brain. Rosenblum et al. evaluated nine patients with the *EPAS-1* mutations and identified a spectrum of vascular malformation [7]. Most patients revealed optic disc elevation without the optic cup, which indicated pseudopapilledema due to congenital vascular malformation. All patients were found to have large retinal veins with abnormal branching patterns by fundoscopy. Some patients had retinal hemangiomatous lesions. Vascular malformations were seen in the brain, neck, and spine, including subarachnoid cavernous malformation, brain parenchymal venous malformation, prominent vein of Galen, and rete mirabile. These patients were neurologically intact, except for some with documented poor vision acuity from early childhood, one patient with a clinically silent frontal lobe venous infarct, and another patient with retinal hemorrhage due to central venous malformation. Rosenblum et al. studied the Epas1A^529V ^transgenic mouse model, which showed similar vascular anomalies and malformations seen in patients, including large dural sinuses, large vein of Galen, and malformation of veins throughout the parenchyma. Mouse models demonstrated the failure of venous regression in the eye and dura. The failure of venous regression, which generally occurs during development in the setting of increased oxygen tension, was proposed as the standard mechanism leading to vascular malformation in this syndrome. This study suggested that somatic *EPAS-1* mutation events occur in an early developmental stage, leading to somatic mosaicism.

In our patient, we suspect the stroke in utero may have been related to abnormal intracranial angiogenesis and/or polycythemia. Although the germline testing was negative for pathogenic mutations, it is not surprising, as *EPAS-1 *mutations associated with Pacak-Zhuang syndrome are mostly somatic mosaicism [2]. The review of the available literature shows only a limited number of cases with germline *EPAS-1* mutation associated with polycythemia and PPGL [11,12]. Wang et al. quantified the frequency of the mosaic mutations in a cohort of patients with *EPAS-1* gene mutations with multiple tissues [13]. It demonstrated that the mosaicism level varies between tissues from the same patient and may be extremely low in some tissues, suggesting the need for multiple samples and techniques to detect mosaicism, as reported by Rosenblum et al. [14].

*HIF-2α/EPAS1* mutation is one of the PPGL mutations that carry the highest metastatic risk (>30%), following *SDHB* (35-75%) and *SDHA* (30-66%) [2]. The emergence of new therapies targeting HIF-2α has been promising. Belzutifan, a selective HIF-2α inhibitor, has been successfully used in an adolescent patient with this syndrome [15]. Recognition of this syndrome is imperative due to the risk of PPGL recurrence and the need for lifelong surveillance. Furthermore, identifying the gene mutation using both germline and somatic testing is crucial for targeted treatment.

The lack of the *EPAS-1 *gene in the Tempus T 648 platform is a limiting factor in diagnosing a somatic mutation with clinical significance in this patient. We hope to raise awareness of the critical role of the *EPAS-1* gene in tumor behavior, risk of disease progression, and recurrence in patients harboring similar phenotypes and carrying these mutations either somatic and/or germline. Another limiting factor we anticipate when testing for a somatic mutation is the variable mosaicism between tissue levels and the importance of multiple sample testing.

## Conclusions

Pacak-Zhuang syndrome should be considered when patients present with early childhood polycythemia, blindness, and hypertension. Screening for PPGL is essential, and genetic testing should be obtained with each case. Identifying a genetic mutation in these patients may be challenging due to somatic mutations of the *EPAS-1* gene and mosaicism. Patients with this syndrome need lifelong surveillance due to a significant risk of recurrence of paragangliomas and their metastatic potential. The treatment with a HIF-2A inhibitor may be a choice in rare cases of metastatic disease. Our patient remains without evidence of metastasis clinically, biochemically, and on imaging surveillance for the past three years.

## References

[1] Chen H, Sippel RS, O'Dorisio MS, Vinik AI, Lloyd RV, Pacak K (2010). The North American Neuroendocrine Tumor Society consensus guideline for the diagnosis and management of neuroendocrine tumors. Pheochromocytoma, paraganglioma, and medullary thyroid cancer. Pancreas.

[2] Nölting S, Bechmann N, Taieb D (2022). Personalized management of pheochromocytoma and paraganglioma. Endocr Rev.

[3] Zhuang Z, Yang C, Lorenzo F (2012). Somatic HIF2A gain-of-function mutations in paraganglioma with polycythemia. N Engl J Med.

[4] Pacak K, Jochmanova I, Prodanov T (2013). New syndrome of paraganglioma and somatostatinoma associated with polycythemia. J Clin Oncol.

[5] Buffet A, Smati S, Mansuy L (2014). Mosaicism in HIF2A-related polycythemia-paraganglioma syndrome. J Clin Endocrinol Metab.

[6] Pang Y, Gupta G, Jha A (2019). Nonmosaic somatic HIF2A mutations associated with late onset polycythemia-paraganglioma syndrome: newly recognized subclass of polycythemia-paraganglioma syndrome. Cancer.

[7] Rosenblum JS, Wang H, Dmitriev PM (2021). Developmental vascular malformations in EPAS1 gain-of-function syndrome. JCI Insight.

[8] Dmitriev PM, Wang H, Rosenblum JS (2020). Vascular changes in the retina and choroid of patients with EPAS1 gain-of-function mutation syndrome. JAMA Ophthalmol.

[9] Fassnacht M, Tsagarakis S, Terzolo M (2023). European Society of Endocrinology clinical practice guidelines on the management of adrenal incidentalomas, in collaboration with the European Network for the Study of Adrenal Tumors. Eur J Endocrinol.

[10] Lenders JW, Duh QY, Eisenhofer G (2014). Pheochromocytoma and paraganglioma: an endocrine society clinical practice guideline. J Clin Endocrinol Metab.

[11] Lorenzo FR, Yang C, Ng Tang Fui M (2013). A novel EPAS1/HIF2A germline mutation in a congenital polycythemia with paraganglioma. J Mol Med (Berl).

[12] Dwight T, Kim E, Bastard K (2021). Functional significance of germline EPAS1 variants. Endocr Relat Cancer.

[13] Wang H, Zhuang Z, Rosenblum JS, Pacak K (2022). Somatic mosaicism of EPAS1 mutations in Pacak-Zhuang syndrome. Endocr Pract.

[14] Rosenblum JS, Wang H, Nazari MA, Zhuang Z, Pacak K (2023). Pacak-Zhuang syndrome: a model providing new insights into tumor syndromes. Endocr Relat Cancer.

[15] Kamihara J, Hamilton KV, Pollard JA (2021). Belzutifan, a potent HIF2α inhibitor, in the Pacak-Zhuang syndrome. N Engl J Med.

